# Nanopriming and Nanocoating as a Next‐Generation Seed Treatment for Enhanced Plant Growth and Pathogen Resistance

**DOI:** 10.1002/pei3.70213

**Published:** 2026-09-23

**Authors:** Okainemen Godfrey Oribhabor, Damian C. Onwudiwe, Vanessa Nessner Kavamura, Jing Ge, Olubukola Oluranti Babalola

**Affiliations:** ^1^ Department of Microbiology, Faculty of Life Sciences University of Benin Benin City Nigeria; ^2^ Soil‐Plant‐Microbe Interactions Group, Faculty of Natural and Agricultural Science North‐West University Mmabatho South Africa; ^3^ Material Science Innovation and Modelling (MaSIM) Research Focus Area, Faculty of Natural and Agricultural Sciences North‐West University, Mafikeng Campus Mmabatho South Africa; ^4^ Department of Chemistry, School of Physical and Chemical Sciences, Faculty of Natural and Agricultural Sciences North‐West University, Mafikeng Campus Mmabatho South Africa; ^5^ Sustainable Soils and Crops, Rothamsted Research Harpenden Hertfordshire UK; ^6^ Institute of Food Safety and Nutrition Jiangsu Academy of Agricultural Sciences Nanjing China; ^7^ Department of Life Sciences Imperial College London, Silwood Park Campus Ascot Berkshire UK

**Keywords:** food security, nano‐agriculture, nanotechnology, seed treatment, sustainable agriculture

## Abstract

In pursuit of sustainable agriculture, current strategies continue to explore environmentally friendly inputs that promote plant development. Seed treatment is an ancient practice used to break seed dormancy and hasten germination. Although traditional seed treatments yield positive outcomes, the methods adopted raise concerns about the negative environmental impacts, such as groundwater contamination from chemical residues. Nanopriming and coating may be more environmentally friendly alternatives for mitigating these problems. Due to their small size and high surface area, and depending on factors such as nanoparticle (NP) type, environmental conditions, and plant species, NPs can efficiently penetrate seeds, promoting nutrient and water uptake and thereby enhancing germination. This review highlights the critical challenge posed by seed dormancy in seed germination and the competitive edge of nanopriming and nanocoating over traditional seed treatment technologies. The impact of nano‐seed treatment on seed sprouting and crop development, as well as the role of nanopores and aquaporins in enhancing seed germination, is discussed. This review also addresses the knowledge gap regarding the mechanism by which NPs enhance seed germination, limited field trial evidence, variability in plant response to NPs and their dual role in simultaneously addressing seed dormancy and conferring protection against seed‐borne pathogens when applied as nanocoats.

## 1 Introduction

Seeds are key components in any plant's life cycle, as they are responsible for the propagation of new plant progenies (Hernández et al. 2024). Food security and sustainable plant production depend on seeds' health. These seed quality characteristics encompass several properties that determine their value and performance. Higher yields and robust plant growth are directly proportional to the use of good‐quality seeds, which demonstrate good vigor and germination rates (Kumawat et al. 2025). Seeds are the foundation of food security for an increasingly populous world. A sufficient supply of healthy, good‐quality seeds is key to human health and nutrition. To fill this gap, seed producers around the world work tirelessly to speed up the development of new seed cultivars (using innovative techniques such as nanopriming and coating) (Munkvold et al. 2025). A complex and crucial physiological aspect of any plant's life cycle is seed germination and this process is sometimes intercepted by biological and environmental stressors, leading to seed quiescence and erratic sprouting (Paul et al. 2022).

The world population is projected to reach 9 billion by 2050, underscoring the urgency of novel solutions to improve food security and grain yields. This is a direct reflection of the quality of seeds sown (Wang, Wu, et al. 2024). Therefore, overcoming seed dormancy and protecting seeds after germination is essential to ensure stable and high crop yields. However, seed dormancy, crop failure after germination (due to pests, pathogens), and nutrient deficiency remain major constraints in crop production and a significant barrier toward achieving Sustainable Development Goal 2 (SDG 2, Zero Hunger). Dormancy is partly induced by environmental factors such as rapid temperature changes, limited water availability, light exposure, and biotic factors such as seed‐borne pathogens (Chandel et al. 2024).

Global crop yield loss due to the ravaging effects of plant pathogens continues to rise, as conventional approaches to disease management have become largely ineffective due to continuous mutation of microorganisms and antimicrobial resistance exacerbated by the abuse of antimicrobial agents. Seeds are susceptible to soil‐ and seed‐borne pathogens that affect quality and ultimately lead to loss of productivity in the field (Liu et al. 2023). Yield loss due to pathogens has been reported to range from 15% to 90% in severe instances (Gebeyaw 2020). Pathogens are also responsible for 20% of disease outbreaks in staple crops such as legumes and rice (Shah and Qin 2025) and result in yearly global economic losses in excess of US$220 billion (Wang, Sundin, et al. 2024). Seed‐borne pathogens cause diseases that hinder germination potential, vigor, and ultimately reduce the economic value of crops. This challenge necessitates the search for pathogen‐free inputs and environmentally friendly antimicrobial agents that can reduce the pathogen burden of seeds in a sustainable manner (Chouhan, Choudhuri, and Mathur 2024).

Traditionally, seed priming has been used to break seed dormancy and enhance sprouting. Farmers have employed this method to promote crop development and overcome several stresses. Priming has also been associated with promoting plant resistance to biotic and abiotic stresses (Paul et al. 2022). Seed priming involves inducing a specific physiological state in crops by treating seeds with chemical or natural agents before germination. This method is an inexpensive, simple, and reliable way to promote germination under stress. Seed priming restarts germination, thereby reducing the germination time and increasing the germination percentage (Mouradi et al. 2023). Seed priming techniques include osmo‐priming (using osmotic solvents), hydropriming (seed immersion in water), chemical priming, hardening priming, biopriming, nanopriming, and solid matrix priming.

While these conventional seed priming techniques are effective under moderate biotic and abiotic stress, they are often ineffective under extreme stress. Innovative approaches, including nanopriming, have been developed to both improve seed tolerance and regulate plant physiological processes in the face of extreme stress, thus strengthening innate resilience mechanisms (Bola et al. 2025). Furthermore, the extensive use of conventional agrochemicals, including chemical seed treatments, increases crop yields but also contributes to environmental pollution. Agrochemicals contain acid radicals that can alter soil chemistry, adversely affect plant health, and increase soil acidity, which may reduce biodiversity. This has prompted research into more sustainable and cost‐effective alternatives to conventional chemical inputs. The consumption of crops with agrochemical residues can cause adverse health effects in humans (Alori and Babalola 2018). Agricultural inputs in nano‐sized form can potentially lower the toxicity posed by conventional seed treatment, which is concentration‐dependent, resulting in improved seed viability (Shelar et al. 2023).

This review focuses on recent contributions that emphasize the role of nano‐sized materials in breaking seed dormancy and coating seeds, thus increasing seed vigor. This review is novel because it addresses the knowledge gaps on the variability in plant response to nanopriming and the paucity of field studies. Beyond laboratory and greenhouse studies covered in most recent reviews, the applicability of this technology in field trials is also reported, contributing to bridging the knowledge gap on current efforts made toward standardizing nano‐seed treatments for positive agricultural benefits, thus promoting the ideals of translational science.

## 2 Overview of Seed Treatment Strategies: The Nanoscale Era

Nanotechnology offers a useful approach that could be explored to ameliorate the challenges associated with crop production that relies heavily on synthetic chemicals for plant development (Fadiji et al. 2022). This technology uses enhanced materials to improve agriculture by maximizing their nanoscale characteristics (Elemike et al. 2019). Recently, the fusion of nanotechnology with agronomic practices has yielded encouraging results, thereby enhancing environmental sustainability and agricultural productivity (Wu and Li 2022).

**FIGURE 1:**
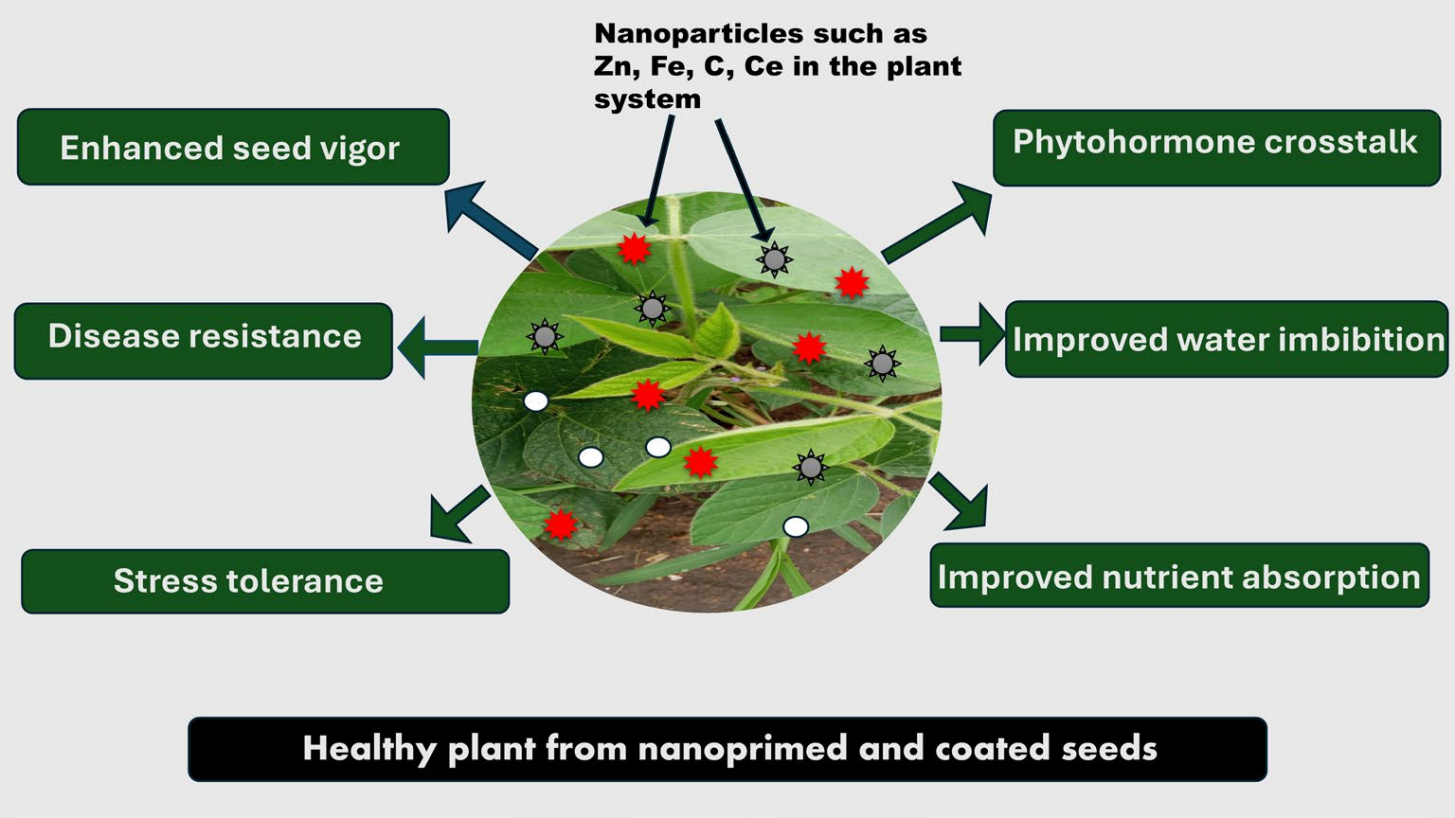
Benefits of nanopriming and nanocoating over traditional seed treatment methods.

Nanopriming has proven to be an economical method to improve crop productivity and promote tolerance to pests and diseases (Salam, Khan, et al. 2022). It involves exposing the seeds to NPs for a short duration, thus inducing molecular and physiological changes, facilitating speedy germination and improving plant resilience and growth (Salam, Afridi, et al. 2022). Nanopriming is simple and cost‐effective, if adopted, this technology can potentially enable smallholder farmers to manage the destructive effects of climate change (Zhao et al. 2024). Nanopriming is considered potentially more efficient compared to other methods of seed priming because, after priming, seeds can repair their metabolism and absorb nutrients more rapidly (Nile et al. 2022). The benefits of nanopriming are highlighted in Figure 1, which include enhanced nutrient uptake, improved disease resistance, enhanced water management, and seed germination. This technology contributes to environmental sustainability, reduces soil water pollution and carbon footprint by reducing the dependence on agrochemicals.

Nano‐seed treatment with different types of nanomaterials has shown marked enhancement in the growth characteristics and germination of seeds for many plant cultivars (Amritha et al. 2021; Chandrasekaran et al. 2020). For instance, the treatment of *Phaseolus vulgaris* L. seeds by Perea‐Vélez et al. (2024) with citrate‐coated (CoFe_2_O_4_) NPs resulted in a marked increase in the crop's potassium concentration by 3%, 13%, and 16% compared with the control. The researchers also noted a 27% spike in the Zn content of the seeds after nanopriming. Similarly, Tamindžić et al. (2023) reported an increase in shoot length, germination energy, chlorophyll content, root and shoot weight, and seedling vigor in pea cultivars after nanopriming with a consortium of NPs (Se, Co, Cu, Mn, Fe, Zn, and Mo).

Zhou et al. (2022) also demonstrated that silver NP‐primed cabbage seeds improved cabbage seedling growth, germination rates, and yield. They also noted a sevenfold increase in Fe content in cabbage leaves due to AgNP seed priming. Similar findings were obtained in green gram seeds treated with several concentrations of zinc oxide NPs. The NPs had a marked positive impact on seedling vigor and germination, especially in the 500 mg/L treatment. The study reported an upregulation in superoxide dismutase (2.86 mg/protein/min) and catalase (2138 units/g) activities after nanopriming. Furthermore, there was a 25.8% improvement in water uptake and an upregulated dehydrogenase activity (0.312 units/g) (Kathiravan et al. 2025).

## 3 Competitive Edge of NP‐Based Seed Treatments

NP‐based seed treatment may present a competitive advantage by enhancing germination, improving stress tolerance, and seedling vigor over conventional seed treatment methods through several unique mechanisms (Table 1). As a result of their high surface area and small size, seed penetration is increased (Durgadevi et al. 2025; Kumari et al. 2026; Yeasmin et al. 2024). NPs can effectively cross biological barriers, facilitating the uptake of water (do Espirito Santo Pereira et al. 2021). They have also been associated with marked improvements in plant germination rates (Ajiboye et al. 2023).

**TABLE 1:** Impact of NPs on seed germination and proposed mechanisms.

NPs	NP dose	Application method	Seed type	Proposed mechanisms	Effects	References
Iron oxide NPs	10–1000 mg/mL	Nanopriming	*Kobresia capillifolia*	An increase in Rubisco activity of the leaves significantly increased the net photosynthetic rate	A decrease in seed mildew rate at 100 mg/L and chlorophyll rate as swollen chloroplasts were observed. Significant antioxidant enzyme activities in the root were recorded	Sun et al. (2023)
Selenium NPs	1–50 ppm	Nanopriming	Tomato ( *Solanum lycopersicum* )	Interaction of selenium NPs with seed protoplastids	Improvement in seed germination by 32.5%. 32.19% decrease in proline content, 21.28% increase in chlorophyll content	Garcia‐Locascio et al. (2024)
Combined Silicon + Titanium + Zinc oxide + Copper oxide + Cerium oxide NPs	SiO_2_, ZnO, CuO, CeO_2_ NPs (50/100 mg/L), TiO_2_ NPs (20/60 mg/L), Fe_3_O_4_ NPs (100/200 mg/L)	Nanopriming	Lettuce ( *Lactuca sativa* )	Reduced accumulation of reactive oxygen species (ROS), malondialdehyde (MDA) in lettuce shoots under cadmium stress	Reduced cadmium translocation in the shoot and roots. Increased antioxidant enzyme activity and chlorophyll content in lettuce	Bano et al. (2024)
Zinc oxide NPs	0–400 mg/L	Nanopriming	Amaranth ( *Amaranthus tricolor* )	The increased seed interaction promoted by the high surface area of ZnO NPs stimulated enzymatic activities, metabolic activation triggered by NPs, and protection against oxidative stress. Induced protein and carbohydrate metabolism, enhanced alpha‐amylase activity and starch hydrolysis during germination.	A 91.5% improvement in germination percentage and seed vigor. Enhanced alpha‐amylase activity (1.9 mg/g).	Geremew et al. (2025)
Silica NPs	1–100 ppm	Nanopriming	Stevia ( *Stevia rebaudiana* Bertoni)	Increased starch metabolism, alpha‐amylase and dehydrogenase activities.	Spike in germination (106%), root, shoot, and seedling dry weight by 283%, 168.9%, and 220%, respectively.	Hasanaklou et al. (2023)
Chitosan NPs	0.1–1 mg/mL	Nanopriming	*Pancratium maritimum* L.	Upregulation of nitrate reductase enzyme activity.	Lowered hydrogen peroxide levels and mean germination time of seeds by 6.85%. Increased water absorption and plumule/radicle ratio.	Allam et al. (2024)

In the age of climate change, ensuring consistent food availability for an ever‐growing global population will require crops to adapt to ecological variation. Although genetic modification has been applied to the development of climate‐smart crops, genetically modified (GM) crops have been poorly accepted by certain societies in developing nations and smallholder farmers. Nano‐treated plants appear to be a better option compared to GM plants (Zhao et al. 2024). Nano‐treatment, over time, has proven to be a useful technique for enhancing the quality of seeds, crop yield and promoting tolerance to diseases (Pachchigar et al. 2022). For example, wheat seeds under drought stress showed improved germination traits after treatment with SiO_2_NPs. The drought amelioration and improved germination were attributed to silicon dioxide NP‐facilitated water uptake. The study also suggested that the small size and increased surface area were key characteristics that enabled the transportation and solubility of SiO_2_ NPs in wheat (Akhtar et al. 2021). In a similar study, a 92% increase in maize seed germination was observed by Naseer et al. (2024) after treatment with ZnO NPs. Sprouting parameters such as root length (26.6 ± 0.9 cm), sugar content (455 ± 9.08 μg/g), proline content (343.71 ± 1 μg/g), number of cobs per plant (33%), chlorophyll content (18.4%), and leaf area (34.3%) were also greatly improved in maize seedlings compared to the control. To further reinforce these improved germination results after nanopriming, Ngwenya et al. (2025) utilized a hybrid of zinc and copper oxide NPs to treat maize seeds before sowing. Their results demonstrated that the ZnO‐CuO‐doped NPs had a significant effect on shoot length, seed vigor index, and the root‐shoot ratio, among other parameters measured. The 125 mg/mL treatment resulted in the highest germination percentage (84%) at 7 days after treatment.

Treatment of seeds with nanomaterials lowers the amount of nanomaterials deposited in the environment and guarantees reduced worker exposure. In line with this concept, zinc oxide NPs of various sizes were applied as a treatment for bitter gourd and red amaranth seeds. The study reported that an improvement in seed germination was dependent on the size of the NPs. A 23% improvement in bitter gourd seed emergence and a 20% increase in shoot length of red amaranth were recorded among other parameters after treatment with less than 30 nm sized zinc oxide NPs (Yeasmin et al. 2024). Thus, nanopriming, with reduced doses and quantities of applied nanomaterials, effectively promoted crop development with reduced input.

### 3.1 Physicochemical Properties of NPs Influencing Their Biological Activities

The method employed in the synthesis of NPs affects their size and shape, which in turn influences their physicochemical properties. Although various physical and chemical methods produce NPs with interesting applications, NPs designed for biological applications are better synthesized using nontoxic approaches involving green materials, such as plants and microorganisms. Eco‐friendly fabrication of nanomaterials with plant extracts provides a more robust alternative for fabricating nanomaterials with distinct physicochemical characteristics and multiple applications (Adeyemi et al. 2022). The small size of NPs enables them to penetrate biological barriers and translocate through a plant's xylem and phloem. Additionally, NPs' increased surface area provides a larger reaction surface, which allows for more biochemical reactions in plants, enhancing assimilation and bioavailability (Azam et al. 2022; De La Torre‐Roche et al. 2020; Guardiola‐Marquez et al. 2022; Saraiva et al. 2023).

Nutrients in their nano‐form are increasingly absorbed by plants, promoting enhanced, efficient nutrient use and availability. Research has shown that the dose of applied nanofertilizers can be 100 times less than that of chemical fertilizers, guaranteeing more efficient nutrient utilization by plants (El‐Bialy et al. 2023; Guardiola‐Marquez et al. 2022; Saraiva et al. 2023; Shalaby et al. 2022; Verma et al. 2022). NPs also promote plant health and defend them against oxidative stress by activating their innate antioxidative metabolites (Adeleke et al. 2022).

Plant species differ in how they absorb NPs because each plant differs in its physiology; as such, they also differ in how they absorb NPs during seed treatment, which influences their growth rate (Khalaki et al. 2020). Recently, various sizes (5 ± 2 nm to 219 ± 2 nm) of zinc carbonate NPs have been used to promote rice growth and inhibit seed‐borne fungi in rice seedlings. The results revealed size‐dependent mycelial growth inhibition of *Fusarium fujikuroi*. Seedling blight was reduced by 82% ± 2%, whereas seed rot was reduced by 81% ± 4%. Seed priming for 8 h increased the germination percentage by 90% ± 2%, overall vigor index (6.84 ± 0.5), and shoot length (9.9 ± 0.6 cm) (Sidhu et al. 2025).

## 4 Nanocoats: An Innovative Stress‐Proof Shield for Seeds

In addition to seed priming, the tradition of seed coating has become a widely adopted method used nowadays to guarantee improved growth (Sohail et al. 2022). Despite the benefits conferred by traditional seed coating methods, many limitations exist, including carrier pollution resulting from the use of recalcitrant coating substances, overuse of agrochemicals, and rising costs of coating materials (Durgadevi et al. 2025; Sohail et al. 2022). Current seed coating studies, such as those involving nano coatings, aim to mitigate these limitations by striking a cautious balance between increased crop production and minimal ecological degradation (Sohail et al. 2022). The need for improvements in seed coating technology is also influenced by factors affecting modern agriculture, such as unpredictable weather and climate change, which are increasingly prevalent and distort seed germination and crop development (Durgadevi et al. 2025).

Nanoscience has transformed seed coating techniques, offering effectiveness and precision (Kiran et al. 2024). The fusion of NPs with seed coatings may guarantee improved nutrient uptake and prevention of the spread of seed‐borne pathogens (Shelar et al. 2023) and limit the effects of stress (Table 2). These NPs lower seed germination time and act as protective shields against seed‐borne pathogens and abiotic stressors, thereby enhancing seed and eventually plant health. They also ensure that seeds are supplied with the necessary nutrients required during the establishment phase (Atanda et al. 2025).

**TABLE 2:** Response of plants to nano‐seed treatment under stress.

Plant	Stressor	NPs	NP concentration	Outcome	References
Jalapeno pepper ( *Capsicum annuum* L)	Drought (Mannitol‐induced)	Zinc oxide and Silicon dioxide NPs	Zinc oxide (100 mg/mL), Silicon dioxide (10 mg/mL)	Increased germination percentage by 25%, Proline accumulation increased 7.5 times, 40% increase in fresh weight of seedlings	Ochoa‐Chaparro et al. (2025)
Peanut ( *Arachis hypogaea* )	Salinity	Graphene oxide NPs	400 mg/mL	Integrity of the plasma membrane was maintained, photosynthesis and antioxidant enzyme activity were enhanced, upregulation of *NRT2, GLU*, and *GLUD1_2* genes in treated stressed plants.12.25% increase in pod yield under field conditions	Yan et al. (2024)
Maize ( *Zea mays* )	Drought, Cold, Salinity	Silver NPs	40 mg/L	Accelerated seed germination, improved seedling vigor under water stress (10% and 20% PEG), salinity stress (50 and 100 mM sodium chloride), and cold stress (15°C). Increased root hair density (17.3%–82.7%). Activation of plant hormone signal transduction. Induction of transcriptomic shift in seeds	Chen et al. (2023)
Spinach ( *Spinacia oleracea* )	Arsenic	Selenium NPs	25–400 μM	NPs treatment notably reduced arsenic accumulation by twofold in roots and 1.5‐fold in leaves, improved photosynthetic efficiency and increased defense enzyme (glutathione peroxidase, ascorbate, and peroxidase) activities	Kumar et al. (2025)
Rice ( *Oryza sativa* )	Salinity	Zinc oxide NPs	200 mg/L	Nanopriming resulted in the highest yield increase (26.96%–36.94%) compared to non‐primed seeds. The proposed mechanisms for salt tolerance were attributed to increased antioxidant enzyme activity and osmoregulatory metabolites	Zhang et al. (2025)
Maize ( *Zea mays* )	*Fusarium graminearum*	Selenium NPs	50 mg/L	Selenium NPs accelerated sugar and starch metabolism in *Zea mays* , induced accumulation of phenolic acid leading to a reduction in disease severity by 26.3% and deoxynivalenol production by 42.5%.	Dong et al. (2026)
Chickpea ( *Cicer arietinum* )	*Fusarium* wilt	Zinc oxide NPs	0.50 g/mL	Nanopriming led to a 90% reduction in disease incidence. Resistance mechanisms were attributed to accumulation of phenols, superoxide dismutase, and increased antioxidant activity	Meerani et al. (2022)
Pearl millet ( *Cenchrus americanus* )	Downy mildew	Chitosan NPs	250 mg/kg	Observed changes leading to upregulation in ammonia lyase, phenylalanine, and polyphenol oxidase gene expression, and higher expression of pathogenesis‐related (PR) proteins 1 and 5. Sixty three percent downy mildew protection modulated by nitric oxide	Siddaiah et al. (2018)
Soybean ( *Glycine max* )	*F. oxysporum*	Graphene oxide/Silicon dioxide/Zinc oxide nanocomposite	50–500 mg/L	The optimal dose (150 mg/L) led to a 57% improvement in fungicidal activity against *F. oxysporum* to ZnONPs, while the composite resulted in a 400% and 196% increase in germination rate and shoot fresh weight, respectively	El‐Zahed et al. (2026)
Wheat ( *Triticum aestivum* )	Karnal bunt disease	Silver and copper NPs	400 μg/mL	A key finding showed that silver and copper NPs induced a marked increase in the expression of PR genes (1 and 6) after 96 h	Jabran et al. (2025)

It has been established that seed treatment is a better approach for ensuring good plant yield than soil amendment. A recent approach to seed treatment involves the use of biodegradable chitosan coats for seeds to improve water solubility and guard seed coat integrity (Ingle et al. 2022; Pirzada et al. 2020). Chitosan nanoformulations have been used as seed coating agents to increase germination rate and biomass accumulation. They have also been utilized as crop growth promoters, as they improve nutrient translocation and other plant parameters (Ingle et al. 2022).

A novel chitosan NPs‐based seed coating was used to coat groundnut seeds. This led to enhanced seedling vigor and germination. Furthermore, structural changes in the treated groundnut seeds were also observed (Vijaykumar et al. 2024). In another study, chitosan doped with histidine was used to create a unique nanoformulation via ionic gelation. The results revealed that the hybrid nanoformulation with unique physico‐biochemical attributes influenced the growth parameters, ROS content, antioxidant enzymes, and chlorophyll content of tomato seedlings. Increased germination percentage (95.9%–99.8%), shoot length (5.42%–25.58%), seedling vigor index (25.90%–42.52%), and root length (14.94%–60.92%) were recorded compared to those of the histidine control (Meena et al. 2024).

Nanotechnology offers a more targeted approach for the delivery of nutrients via seed coatings (Durgadevi et al. 2025) compared to traditional fertilizers, which often leach and run off when applied to the soil, causing environmental pollution and nutrient loss. These inefficiencies are reduced when nanofertilizers are introduced as seed coatings, enhancing the transportation of nutrients within seeds at the early stages of germination (Yadav et al. 2020). Furthermore, NPs support slow nutrient supply within plants and the stabilization of phytohormones (Oribhabor et al. 2025). Abeywardana et al. (2021) showed that seed coatings made of urea hydroxyapatite and zinc, more precisely, translocated nutrients and improved plant nutrient utilization.

NPs have recently been used to encapsulate fungicides, creating more environmentally friendly seed treatments for the control of *F. fujikuroi* (seed‐borne fungus of rice). Zinc NPs coating was used as a transport vehicle for the fungicide, ipconazole. Nanocoating with zinc NPs effectively controlled the proliferation of *F. fujikuroi* spores by deeply penetrating rice and fungal hyphae, thus promoting rice growth. The germination rates increased from 95.33% to 96.00%. The study concluded that treatment with a hybrid zinc nano carrier improved the rice root length, fresh weight, and seedling emergence rate after 35 days of sowing (Zhang et al. 2024).

### 4.1 Interaction of NPs With Seed Coats: The Role of Nanopores and Aquaporins

Nano‐formulations improve seed health and the sprouting rate through precise delivery systems that allow nanomaterials to interact long enough with seed coats, facilitating the absorption of necessary nutrients (Atanda et al. 2025). When applied as a seed coating, NPs either penetrate the seed tissues or adhere to the seed pericarp. However, there is limited knowledge on the mechanism by which NPs penetrate seeds, which may vary depending on NPs and seed properties (Gohari et al. 2024). Researchers have proposed several pathways by which nanomaterials can enter seeds. NPs may diffuse directly into the seed via the coat (Eevera et al. 2023). They may also penetrate through microscopic holes present in the seed coat and deposit in the cells of the embryos (Faizan et al. 2024). Furthermore, ion channels and aquaporins in the cell membrane of seeds may pave the way for nanomaterial absorption. Although these channels typically allow for the movement of water and ions, they may also enable nanomaterials to flow through (Li et al. 2020).

Nano coating also stimulates aquaporin gene expression, thus enhancing water uptake across biological membranes (Aggarwal et al. 2025). Through this technique, NPs interact with the seed coat, creating nanopores, promoting the breakdown of starch by stimulating antioxidant processes in seeds and ROS production, resulting in accelerated seed germination (Donia and Carbone 2023).

In addition to nanopore and aquaporin creation, other mechanisms responsible for the effectiveness of seed germination after nano‐seed treatment include: restarting the seed's antioxidant/ROS system and producing ‐OH radicals that loosen the cell wall (Pachchigar et al. 2022). The type and properties of NPs affect their interactions with seed coats and how they are translocated within the seed. Smaller NPs (> 30 nm) penetrate the seed coat more easily. Furthermore, plant species differ in their receptiveness to NPs absorbance (Faizan et al. 2024).

## 5 The Role of NPs in Defending Plants Against Pathogens

Viable seeds form the pillar of the agricultural system. This is important because of the high yield loss recorded in globally relevant crops such as wheat, rice and maize due to climate change and the destructive effects of pathogens and diseases affecting plant yield (Ajilogba et al. 2021; Chouhan, Choudhuri, and Mathur 2024). Plant pathogens, which are pests that cause diseases in plants (Oribhabor and Iyekekpolor 2023), pose serious threats to agriculture‐dependent countries, as they can cause major economic losses that are capable of crippling the economy.

Seed‐borne pathogens continue to attack seeds, reducing crop yields each year (Chouhan, Choudhuri, and Mathur 2024). Several reports have demonstrated the usefulness of chemical pesticides in reducing disease severity in agricultural systems (Oyedoh et al. 2025). However, these chemicals cause harm to the environment. Recent research aims to provide eco‐friendly, sustainable alternatives for pathogen control (Fadiji et al. 2022).

Nanotechnology provides efficient tools that can bypass traditional agro‐practices and curb the ravaging effects of pathogens sustainably (Chouhan, Mathur, et al. 2024), including seed‐borne pathogens. The use of nanotechnological tools can be beneficial to farmers by helping them reduce crop losses by using NPs to protect crops from diseases (Fadiji et al. 2022) and preserve seeds during storage. Seed‐borne pathogens cause seed abortion, rot, and necrosis and can also cause disease at the later stages of crop development. Even small seed‐borne inoculum can cause severe yield loss and enable the transport of pathogens to virgin environments, where they can cause disease in the same or a different crop. Seed contamination could lead to trade barriers between countries in a bid to protect local production (Moumni et al. 2023).

**FIGURE 2:**
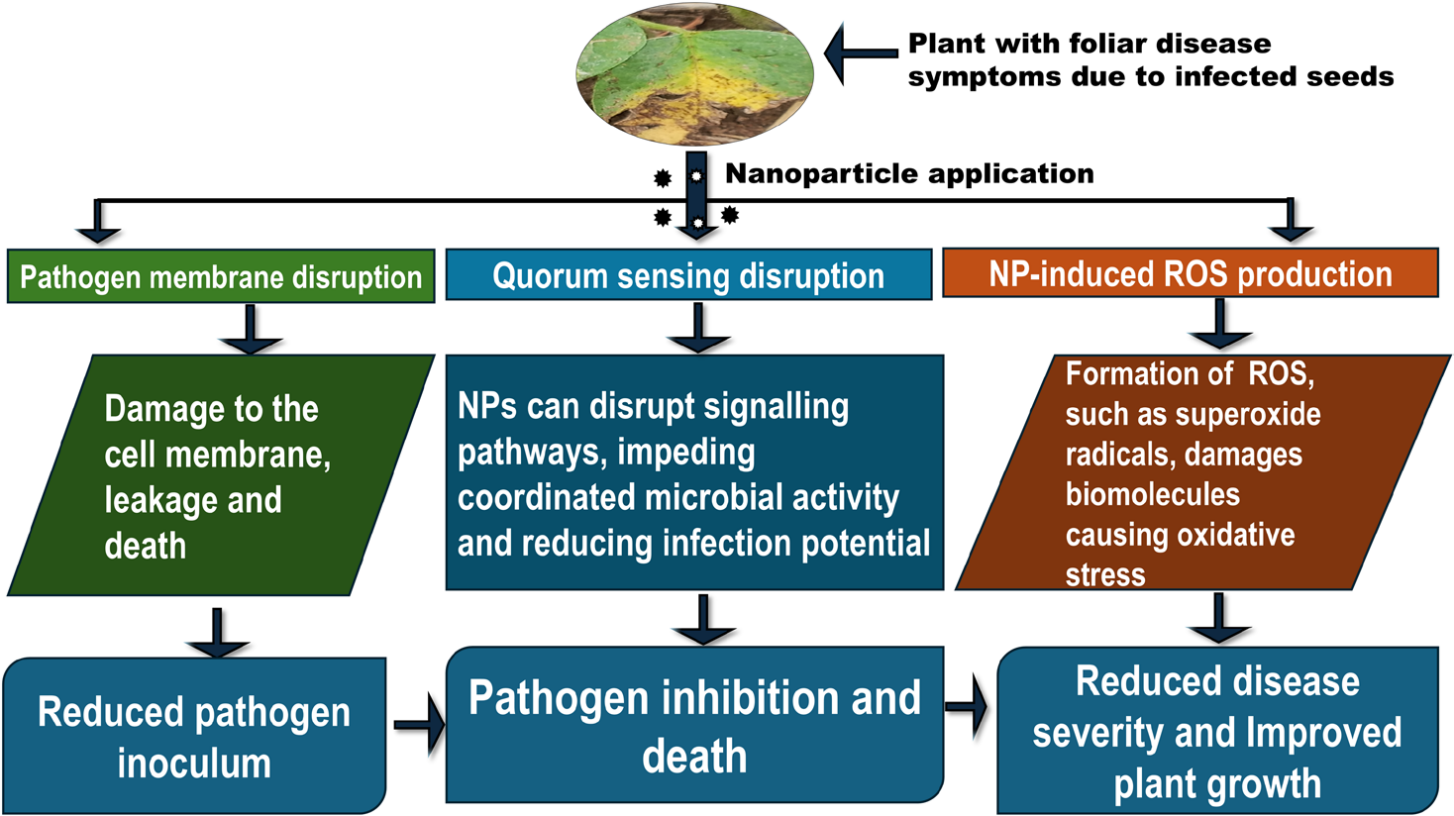
General antimicrobial mode of action of NPs.

Nano‐seed treatment protects seeds from seed‐borne pathogens by inducing nanopore formation, enhancing water translocation and upregulating antioxidant mechanisms. This technology also triggers starch metabolism by stimulating amylase production, leading to improved seed emergence. Furthermore, nanocoating and priming induce ROS that act as a key signaling cue for several events associated with metabolite production and stress tolerance (Sarmah et al. 2024) (Figure 2). It is important to note that the antimicrobial action of NPs depends largely on shape, size, synthesis route, plant type, and environmental factors. Different nanomaterials will elicit varied responses depending on these factors.

Furthermore, the mechanism by which NPs‐mediated translocation protects seeds may also involve passive diffusion of nanomaterials and endocytosis, leading to physiological changes and alleviation of biotic stress (Prakash et al. 2025). Mechanistically, nanomaterials are regarded as plant regulatory molecules because they can modulate an array of biochemical and physicochemical processes, stress‐related gene expression, and the plant defense system (Singh, Choudhary, et al. 2024). Also, the activation of stress‐responsive genes, transcriptional reprogramming, ion homeostasis, activation of Gamma‐aminobutyric acid (GABA) shunt, and the modulation of signaling pathways are some of the molecular mechanisms that are reportedly associated with stress mitigation after nano‐seed treatment (Al‐Quraan et al. 2023; Zhang et al. 2026).

Buttressing these facts, Muhae‐Ud‐Din et al. (2025) ascribed the improved resistance of chili to *Colletotrichum capsica* to improved mycelial permeability and upregulation of defense genes such as CaPDF 1.2 and CaPR‐5 in the plant after treatment with silver NPs.

According to Ghimire et al. (2023), 120 pathogens affect wheat, 42 of which are seed‐transmitted. The production of wheat, one of the world's top five cultivated crops, is severely hindered by seedling disease. To curb this growing menace, Chouhan, Mathur, et al. (2024) used Ag + nanochitosan for nanopriming seeds for the management of *Aspergillus niger* and *A. flavus* . Their report revealed complete inhibition of mycelial growth for both fungi at a 0.5 mg/mL NPs concentration, alleviating fungal stress and improving seed germination. A remarkable decrease in disease incidence to 10% was observed compared with that of the control (88.66%). Scanning Electron Microscopy (SEM) revealed structural changes in the membranes of NPs‐treated fungi spores. NPs treatment further increased albumin, gluten, gliadin, total phenol, and starch content in wheat seeds.

Molybdenum (Mo), a micronutrient for plants, was recently doped with tungsten (VI) oxide (WO_3_) NPs to produce a nanomaterial with enhanced antibacterial activity against *Xanthomonas oryzae* and *Pseudomonas aeruginosa*, disease‐causing organisms in kodo millet. The results from this study show that Mo‐doping enhanced the antibacterial activity of WO_3_ NPs. The enhanced antibacterial activity was ascribed to the strong association of the NPs with the bacteria lipid groups, leading to interference with membrane proteins, increased ROS production and cell death (Antony et al. 2023). Similarly, zinc sulfide nanospheres were recently used as nanoformulation against rice seedling blight and rot. The nanoformulation demonstrated an eightfold antimicrobial activity compared to the chemical fungicide, carbendazim. The antifungal mechanisms of action postulated include cell wall and membrane disruption, protein leakage, hyphal death and inhibition of certain enzymes (1,3‐β‐glucan synthase and 1,4α‐demethylase). The treatments reduced the disease incidence of seed rot and seedling blight by 62.59% and 62.59%, respectively. In addition, the nanoformulation boosted seedling growth (Khepar et al. 2022).

A unique advantage of nanocoating is the ability of nanoformulations to protect seeds during storage. Nanocoating forms a barrier over seeds, extending their shelf life by conserving water and reducing susceptibility to pathogens, thus maintaining germination potential and seed vigor over time (Atanda et al. 2025). Recently, a green strategy for mung bean protection and long‐term storage was proposed by Ji et al. (2023). Their study showed that coating mung beans with photothermal, biocompatible polydopamine NPs and a cellulose derivative (Cell‐N^+^) eradicated *Aspergillus flavus*, an aflatoxigenic fungus, when coupled with photothermal therapy. In another study, a hybrid composite (ZnO‐CuO NPs) was applied as a seed treatment against aflatoxin B1‐producing *A. flavus* in maize seedlings. A 13.1% inhibition against the fungus was observed at the 0.5 ppm dose. Additionally, the nanoformulation improved seed germination with a final germination percentage of 90.7% (Ngwenya et al. 2025). Sidhu et al. (2026) reported the synthesis of zinc (II)‐salicylate NPs and demonstrated that treatment of rice with this NP led to a significant reduction in seed rot by 16.5% and seedling blight by 10.5% caused by *Fusarium verticillioides*. The authors attributed the antifungal activity and improved seedling germination (80%) after nanocoating to enhanced zinc bioavailability and the combined effect of salicylate ligand and zinc ion. Clearly, NPs penetrate seed coats, increase water absorption due to their small size and upregulate aquaporin genes, thus improving seed germination and reducing the adverse effects of stress (Shelar et al. 2023).

## 6 Nanopriming for Plant Growth: Current Status

Nanopriming continues to gain scientific attention, especially in the context of modulating seed signaling pathways and metabolism, to produce seedlings with improved germination attributes (Ghosh et al. 2024). This is a promising field in phyto‐nanotechnology that can equip treated seeds with stress tolerance and relieve oxidative stress in crops (Mouradi et al. 2023). Effective seed germination promotes the successful establishment of plants, and nanopriming may potentially prove, as earlier established, to be more effective than other seed priming techniques because of the distinct properties of NPs (Khalaki et al. 2020).

The transformative effect of nanopriming on seed development and plant growth was investigated by Hemalatha et al. (2024). Their study aimed to determine the impact of silver and copper NPs on the sprouting attributes of Green gram seeds. Their findings revealed that silver NPs improved germination by 81.67%, while copper NPs resulted in a 79.33% improvement at the 1000 ppm concentration. After storing the seeds with these NPs for 9 months, a marked increase in the biochemical parameters (catalase and alpha‐amylase levels) of the seeds was observed. Additionally, priming alfalfa with lanthanum oxide NPs (La_2_O_3_NPs) reportedly improved salt tolerance and seed vigor. NPs improved the germination rate (30.0%–81.3%), seedling length (8.6–13.3 cm), and root length (8.3–23.4 cm). Some mechanisms proposed to explain salinity tolerance include: penetration of alfalfa cotyledons by La_2_O_3_NPs, alteration of the seed coat, increased alpha‐amylase activity, decreased H_2_O_2_, and enhanced water absorption (Gao et al. 2025).

Cell division in sprouting seeds starts after the emergence of the embryonic root. Seed priming, which prolongs the time span of the 2nd stage of seed sprouting, is concluded before radicle emergence. Thus, seed priming prepares seeds for the subsequent stage of cell division, even if it does not directly influence it (Aggarwal et al. 2025). Upon priming selected cultivars of *Capsicum annuum* L. seeds with Cu, Ag, and Cu‐Ag NPs, Mawale et al. (2024) reported an improvement in the seedling vigor index (2494–3112.66), germination (96%), and biomass (46%). Recently, seed treatment with carbon‐based nanomaterials has been beneficial for agrosystems (do Espirito Santo Pereira et al. 2021). Nano‐carbon seed treatment can be utilized to promote germination, improve seed protection during storage, and support crop development (López‐Vargas et al. 2020; Zhang et al. 2022).

Carbon‐based nanomaterials, like other NPs, offer competitive advantages over conventional priming techniques as a result of their small size, enabling them to penetrate seeds easily (Yan et al. 2023). Additionally, they can be doped with metal NPs with antimicrobial properties to provide enhanced defense against pathogens (Fonseca et al. 2024). After priming pearl millet seeds ( *Pennisetum glaucum* ) with carbon nanotubes, an increase in germination percentage (80%) was observed by Sharma et al. (2023). The positive effect was dose‐dependent (the optimum dose was 90 ppm), as higher concentrations (120 and 150 ppm) led to a decreased germination percentage. The toxicity at higher concentrations was linked to the blocking of the seed coat by graphene sheets, leading to reduced water permeability. The possible mechanisms responsible for the NPs' effects on the seeds are the release of ROS, apoptosis, reduced transpiration rates and increased lipid peroxidation and protein carbonylation.

### 6.1 Plant Defense and Stress Memory Activation via Nanopriming

Omics technologies aid the understanding of seed priming procedures, the genes associated with DNA methylation, and translation initiation factors. Understanding the molecular and biochemical mechanisms that create the priming effects on seed sprouting and the defense systems that are involved could facilitate the identification of biochemical and molecular markers for several priming regimens (Corbineau et al. 2023). A key molecular “imprint” on plants after nanopriming is stress memory. This is an improved tolerance that nanopriming induces in plants after nanopriming, enabling the plants to respond more efficiently to future stress exposure. This stress memory is enabled through epigenetic, transcriptional, and translational changes in the plant, resulting in sustained tolerance (Gohari et al. 2024).

For example, Rai‐Kalal et al. (2021) established in their study using zinc oxide NPs to combat drought stress in wheat that after seed priming, a stress memory was activated, allowing for improved tolerance during subsequent drought stress exposure. The study proposed that the subsequent stress tolerance was associated with the role of H_2_O_2_, which acted as a signaling molecule, resulting in lowered levels of reactive oxygen species production in stressed plants, indicating improved tolerance to drought. Similarly, nanoceria coated with polyacrylic acid priming was shown to initiate stress memory in the model plant, *Arabidopsis*, which was transferred to subsequent generations. The study reports an induction of DNA methylation changes in the offspring and improved transgenerational salinity tolerance. Nanopriming was also shown to improve POD activity by 15.69% in the offspring (Li et al. 2025).

Nanopriming enhances plant defense by improving the expression of stress proteins and genes, antioxidant molecules, and signaling metabolites, which lowers the energy demands of plants during adverse conditions (Kumar et al. 2022).

It is noteworthy that most reported successes associated with seed defense from biotic and abiotic stressors have emanated from laboratory and greenhouse trials. It is important to conduct extensive field trials to validate the potential of this technology, as well as optimum dosage, toxicity, and the influence of environmental factors on nano‐seed treatment before commercial adoption. However, a limited number of studies have reported the response of plants after nano‐seed treatments in field trials, and some of these outcomes are presented in Table 3.

**TABLE 3:** Response of plants in field trials after nanopriming.

Plant	NPs	NPs concentration	Duration of study	Outcome	References
Durum wheat	Silver NPs	10–80 ppm	2 years (Two seasons)	40 ppm produced the best results for productivity (10.453 tons/ha), straw, grain yield, and 1000‐g weight by 41%, 46%, and 5%, respectively	Al Salama et al. (2025)
Bitter gourd ( *Momordica charantia* )	Zinc oxide NPs	50–100 ppm	45 days	Seeds primed with 100 ppm NPs for 48 h resulted in a 29% germination increase. Similarly, marked increases were recorded in parameters such as fruit number (41%), fruit length and density (29%), and fruit diameter (42.3%), compared to the controls	Mazhar et al. (2024)
Wheat	Silica NPs	200 mg/mL	4 months	NPs treatment led to a 27.4% increase in tillers and 66.7% elongation of internodes. Furthermore, an increase in the expression levels of key genes such as *CHLH* and *POR* for chlorophyll, *SPS*, and *SUS* for soluble sugars, was observed	Li et al. (2023)
Pigeon pea ( *Cajanus cajan* )	Iron and Zinc NPs	50–100 ppm	Two planting seasons	The study highlights the superior performance of green‐synthesized iron and zinc NPs over commercial products. It further reports that both NPs demonstrated a 77.35% increase in stalk yield, 77.41% higher seed yield (1728 kg/ha), and a 52.20% higher husk yield (828 kg/ha)	Kurdekar et al. (2025)
Rice	Nanoscale zero‐valent iron (nZVI)	10–80 mg/L	2 years	Nanoprimed seeds produced plants with increased tiller number (2‐fold higher), broader leaves and enhanced biomass compared to the control. 20 mg/L produced a 3.8‐fold increase in yield in both years. Also, enhanced starch, lipid, thiamine, and ascorbic acid were observed in treated grains	Guha et al. (2022)
Onion	Gold NPs	5.4 ppm	2 years	A significant change in emergence (63.2%) was observed compared to the control (37.4%) in both years. Similarly, compared to the control, a 23.9% yield increase was observed in gold NPs‐primed onions	Acharya et al. (2019)
Rice	Zinc oxide NPs	25–100 ppm	90 days	25 ppm nanopriming resulted in the best growth, evidenced by enhanced seedling vigor, shoot, and length. 25 ppm priming coupled with 100 ppm foliar spray significantly improved antioxidant enzyme activity, chlorophyll, and protein parameters. An upregulation of zinc transporter genes such as OsNAAT and OsZIP1.1 was similarly observed in the root tissues	Gamit et al. (2025)
Maize	Magnesium NPs	37.5–600 mg/L	2 seasons	Seed treatment shortened the V4 and V5 phenological stages by 4–5 days, leading to an increase in grain yield by 38% and 57.1% at the 150 and 300 mg/mL doses, respectively	Segatto et al. (2023)
Cluster bean ( *Cyamopsis tetragonoloba* L.)	Silicon NPs	50–400 μg/mL	2 seasons	The study showed that silicon NPs priming was more effective than bulk silicon, especially the 200 μg/mL concentration. The nano‐treated seeds produced the highest germination percentage (94.67%), plant height (132.67 cm) and a 258% rise in grain yield compared to the control	Kumari et al. (2026)

Certain challenges militate against the commercial adoption of this technology at market scale. These include limited knowledge of the long‐term effects on the environment, soil microbiome and sentinels, uncertainty regarding the degradation rate of different nanocoats, high upfront cost, limited regulatory framework, concerns about bioaccumulation, especially of nanomaterials from heavy metals such as silver and gold, and public perception.

Other challenges that prevent the successful transition of the positive potential outcomes of agricultural nanotechnologies from laboratory and greenhouse trials to field‐scale evaluation and ultimately to commercialization include a lack of efficient delivery at field scale, scalability problems, differences in standardized guidelines between regions such as Europe, the Americas, and Asia, and synthesis reproducibility (Ullah et al. 2026). Also, Shelar et al. (2023) opined that the successful implementation of these nano‐enabled seed treatment strategies will largely depend on the cooperation of researchers, government agencies, and communities. The authors also recommended extensive screening and optimization in relation to different seed types and consistent legal frameworks as some of the actionable steps toward commercialization. Similarly, Xiao et al. (2026) proposed the development of systematic field validation strategies and a ‘One Health’ assessment framework to holistically evaluate the risks.

## 7 Translocation and Uptake of Nanomaterials in Plants

**FIGURE 3:**
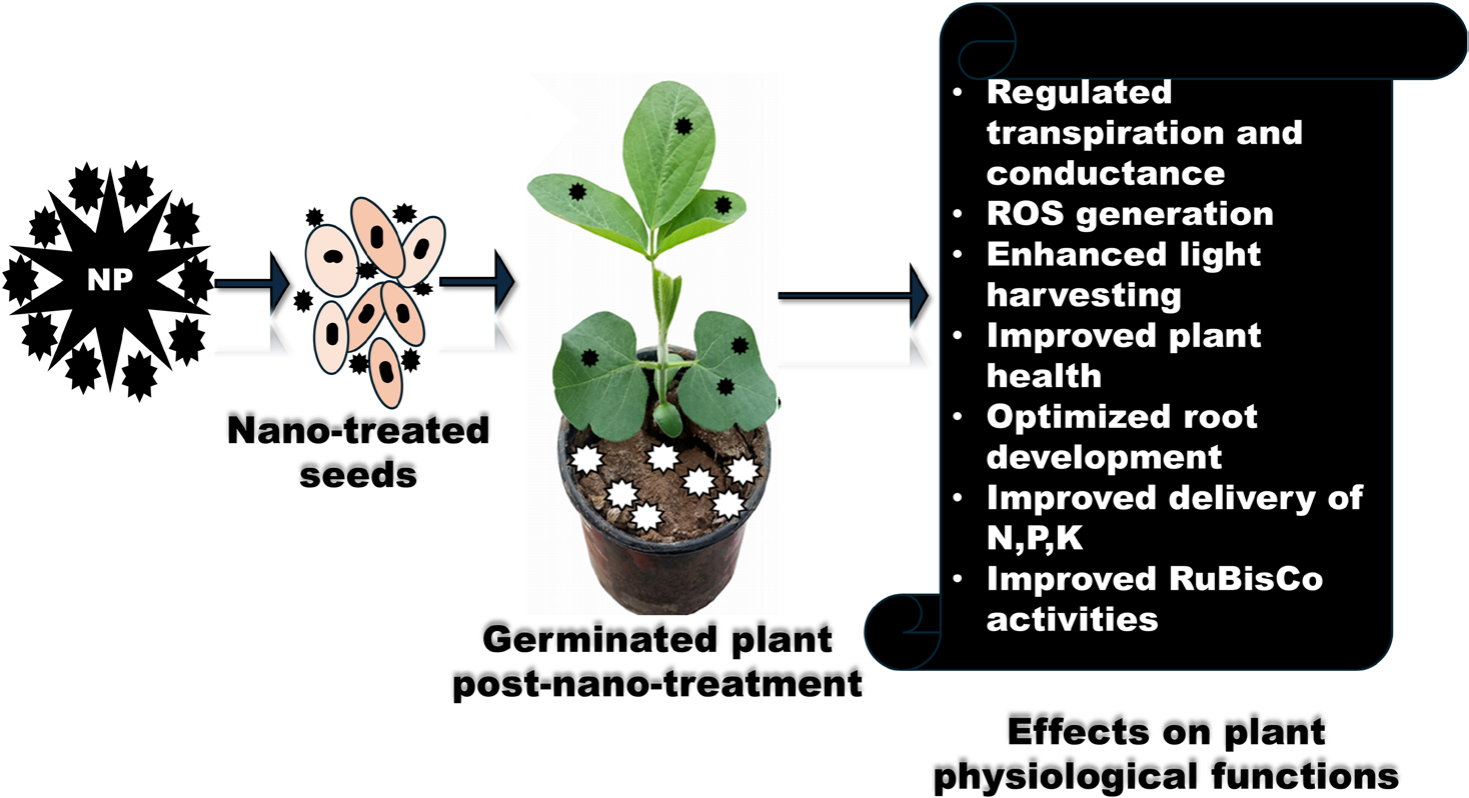
Effect of nanopriming and coating on plants' physiological, metabolic and biochemical functions.

NPs induce the translocation of water and nutrients in plants. They can enter plants via seeds, roots, shoots, and leaves (Figure 3). They are subsequently translocated through processes such as diffusion, phloem loading, and bulk flow (Khan et al. 2025). Several attempts to improve nutrient assimilation and crop biofortification have been largely unsuccessful. Thus, nanotechnological approaches should be explored (Elemike et al. 2019). The use of nanotechnology in farming systems has demonstrated the capacity to reduce nutrient loss and enhance crop production (Khan et al. 2025).

Understanding the biological impacts of NPs on plants and their translocation and uptake is necessary to gain insights about their environmental consequences and optimize their utilization in farming practices (Prakash et al. 2025). The effectiveness of nanopriming is directly correlated with how well NPs penetrate the seed, breach its biological barrier, and translocate within the plant. Metal oxide NPs increase sprouting rates in several crops by promoting energy production and enzymatic activities in seeds (Guo et al. 2022). The nanoscale size of these materials gives them an edge, as their enhanced seed coat penetration facilitates cellular respiration and nutrient mobilization (Singh et al. 2021).

Titanium oxide NPs, for instance, were shown to improve the breakdown of stored starch and enhance water uptake in seeds, thereby accelerating germination (Shah et al. 2021). Additionally, during NPs uptake by seeds, a sizable amount remains on the seed surface. This coating confers many benefits, including stimulation of aquaporin genes and an elevation in the plant sugar content, promoting seed emergence and performance.

Dose and size constitute key NPs properties that influence their translocation and uptake. NPs composed of the same constituent, with varying sizes and concentrations, may display different physiological behavior in crops (Prerna et al. 2021). Tombuloglu et al. (2024) investigated the impact of size on the uptake and translocation of iron oxide NPs in barley. NPs with average sizes of 10 and 100 nm were applied to barley at different doses at the sprouting and seedling stages. The findings showed that although NP size had no notable impact on seedling emergence, it did enhance barley's germination attributes. NP translocation also increased the carotenoid and chlorophyll contents of the plants. The study concluded that a spike in iron content was observed in roots and leaves with increasing NP concentration, indicating that NPs applied as seed priming agents translocated from the roots to the shoots.

In addition to the root‐to‐shoot translocation of NPs, studies have shown that NPs can also translocate from shoot‐to‐root, promoting crop development and ending up in the plant's rhizosphere, improving the microbiota richness (Xu et al. 2023). Li et al. (2022) reported a notable increase in the abundance of rhizosphere microorganisms, including Alpha proteobacteria, Delta proteobacteria, Bacteroidetes, and Gemmatimonadetes, after treating pepper plants with selenium NPs. Nanotechnology, thus, provides a platform for the sustainable transport of nanoscale nutrients to crops, utilizing their nanoporous surfaces, which improves the translocation and accessibility of nutrients (Chugh et al. 2022).

### 7.1 Factors Influencing NPs‐Plant Response

Different NPs translocate within plants and produce variable responses depending on factors such as plant species, the kind of permeable membrane each plant possesses, dose (which could be phytotoxic or tolerant), particle size, route of administration (priming, foliar spray, or soil drench), and environmental conditions such as soil properties.

According to Ahmed et al. (2021) factors influencing the beneficial or phytotoxic effects of NPs on plants are the species and growth stage of the treated plant, time of exposure and physicochemical properties of the tested NPs such as concentration and particle size. Similarly, Matras et al. (2022) revealed that surface charge influences NPs' activity. Their study evaluated the toxic effect of three variants of silver NPs with different surface charges on two monocots and dicots. It was reported that positively charged silver NPs had a greater inhibitory effect on shoot dry mass and photosystem II efficiency, compared to negatively charged silver NPs.

NPs size also influences their effect on plant development. Smaller sized nanomaterials may have an increased surface‐to‐volume ratio, making translocation easier compared to large‐sized NPs, which can aggregate and block the plant's phloem and xylem. For example, it has been reported that the lowest dose and smallest grain size of Ag‐TiO_2_ NPs better promoted the growth of spinach seeds (Gordillo‐Delgado et al. 2020). Other parameters such as dissolution rate, soil mobility, pH, and aggregation state also influence the activity of the nanomaterial and the plant response (Hassan et al. 2026).

The translocation of NPs is also plant‐type dependent. Zhang et al. (2019) compared the translocation of nanoceria in four different plants. The study highlights that the translocation mechanism correlated with the presence or absence of phosphate in the nutrient solution. The study also highlighted the composition of the root exudates and plants' xylem as key determinants of nanoceria translocation and transformation within the plants.

The limitation of nanotechnology in seed treatment is that NPs, alongside their promising effects, may pose a risk to the food chain, causing changes in metabolic processes. The translocation of nanomaterials may potentially change the pattern of plant proteins, such as those involved in cellular functions like redox metabolism and energy regulation (Farooq et al. 2024). Hence, the urgent need for extensive field trials to determine the long‐term characteristics of these materials under changing environmental conditions and plant types.

## 8 Challenges, Fate of Nanomaterials in Soil–Plant–Water Systems

The physical and chemical diversity of soil particles makes it difficult to evaluate the toxicity of nanomaterials through the way they interact with the soil, as NPs interact with different soil textures in varied ways, depending on certain factors such as the presence of soil organic matter or preexisting waste (Alhassan et al. 2024; Xuan et al. 2023). Recent overuse of nanomaterials has made it critical to understand their fate in both living hosts and the environment (Zafar et al. 2023).

The physicochemical attributes of a nanomaterial will determine the amount that leaches into the soil. Due to their high mass‐to‐surface‐area ratio, NPs can be immobilized by binding to the soil particles (Santos et al. 2023). Furthermore, NPs may be absorbed by microorganisms in the soil, creating unplanned consequences for the crop ecosystem (Goyal et al. 2025). A limited number of studies exist on the interaction between plants and NPs due to limited technical knowledge of the strategies involved. To garner broader acceptance, holistic risk assessment, and biocompatibility studies are needed to ascertain the fate of nanomaterials in the soil–plant system (Chugh et al. 2022).

The impact of nanomaterials on water systems has also garnered attention, as nanomaterials may seep into groundwater when applied in agriculture, or runoff into nearby water bodies, posing a threat to aquatic life (Singh, Thakur, and Kumar 2024). Therefore, it is important to understand the mechanisms of NPs accumulation and translocation in the environment and evaluate the potential adverse impact on plant development (Tirkey et al. 2025), the knowledge of which is largely lacking, possibly due to the expensive analytical technologies required to efficiently detect, characterize, and quantify nanomaterials in the environment. It is important to develop affordable analytical tools that are robust for detecting nanomaterials in plant and soil systems (Hendricks et al. 2023).

## 9 Future Directions and Conclusion

The application of nanotechnology in sustainable agricultural practices is becoming a well‐established process. As knowledge of nanotechnology's usefulness in farming practices has improved, more efforts should be made to understand the long‐term impacts of NPs on the ecosystem. Most positive nanopriming and nanocoating outcomes emanating from controlled laboratory experiments and greenhouse studies, although hopeful, still require validation in real‐world settings where climatic and environmental factors come into play. The fate of nanomaterials in plants and their long‐term impacts on the soil microbiota need to be studied. The shape and size of NPs influence their biological activities. More studies should focus on understanding how nanomaterials' shape and size influence seed penetration and antimicrobial activity. Additionally, a good mechanistic understanding of the association between NPs and aquaporins at the omics level should be considered. It is also important to understand NP translocation within plant tissues to guarantee safe food. Future studies should focus on high‐tech microscopy and ROS quantification to validate the proposed antimicrobial mechanisms. Also, toxicity and biocompatibility assays should be developed to establish safe doses (El‐Zahed et al. 2026).

The effects of nano‐seed treatment on plant viruses and nematodes are largely understudied, as most research has focused on plant pathogenic bacteria and fungi. An understanding of the effects on these neglected groups of organisms will provide knowledge on the holistic impact of nanomaterials in defending plants against a variety of pathogens. Furthermore, the many benefits of nanopriming and nanocoating for sustainable agriculture need to be communicated to agricultural stakeholders to discourage continued reliance on chemical seed treatments. More regulatory acts should also be enacted, ensuring strict adherence to good nano‐formulation manufacturing standards, optimizing the dose and reducing phytotoxic risks both to treated plants and nontarget species. While conventional seed treatments may pose environmental concerns, the long‐term environmental fate, persistence, and ecotoxicological effects of NPs remain insufficiently understood and should be looked into in future studies.

The quality and health of any plant directly correlate with those of its seeds. As seeds face a myriad of stressors and pathogens continue to mutate, scientists should develop stealth nanomaterial coatings that evade pathogen detection systems, ensuring the effective delivery of antimicrobial formulations. Additionally, the current developments in machine learning and artificial intelligence should be harnessed to develop algorithms that enable smart nanocarriers for programmed nutrient dissipation during seed treatments. These nanocarriers can also be programmed to release chemicals and signals for effective real‐time seed monitoring.

In this review, we have established that nanopriming and nanocoating offer an eco‐friendly seed treatment method, improving germination, promoting pathogen resistance, and advancing sustainable agriculture. Current gaps in knowledge have been summarized, and a clear understanding of the efficient utilization of nutrients through these technologies has been reinforced, thereby promoting reduced dependence on chemical inputs. This study provides a template for future research that promotes food security and eco‐friendly agricultural practices. Priority research directions, such as standardized NPs characterization, long‐term environmental monitoring, and regulatory frameworks, should be given serious attention.

## Funding

This research was funded by the International Centre for Genetic Engineering and Biotechnology (ICGEB) (Grant number: CRP/ZAF22‐93) awarded to O.O.B.

## Conflicts of Interest

The authors declare no conflicts of interest.

## Data Availability

No new data were generated. Data sharing does not apply to this article.
